# MirGeneDB 2.1: toward a complete sampling of all major animal phyla

**DOI:** 10.1093/nar/gkab1101

**Published:** 2021-11-25

**Authors:** Bastian Fromm, Eirik Høye, Diana Domanska, Xiangfu Zhong, Ernesto Aparicio-Puerta, Vladimir Ovchinnikov, Sinan U Umu, Peter J Chabot, Wenjing Kang, Morteza Aslanzadeh, Marcel Tarbier, Emilio Mármol-Sánchez, Gianvito Urgese, Morten Johansen, Eivind Hovig, Michael Hackenberg, Marc R Friedländer, Kevin J Peterson

**Affiliations:** The Arctic University Museum of Norway, UiT- The Arctic University of Norway, Tromsø, Norway; Science for Life Laboratory, Department of Molecular Biosciences, The Wenner-Gren Institute, Stockholm University, Stockholm, Sweden; Department of Tumor Biology, Institute for Cancer Research, The Norwegian Radium Hospital, Oslo University Hospital, Oslo, Norway; Institute of Clinical Medicine, University of Oslo, Oslo, Norway; Center for Bioinformatics, Department of Informatics, University of Oslo, Oslo, Norway; Department of Pathology, Institute of Clinical Medicine, University of Oslo, Oslo, Norway; Department of Biosciences and Nutrition, Karolinska Institute, Huddinge, Sweden; Department of Genetics, Faculty of Sciences, MNAT Excellence Unit, University of Granada, Granada, Spain; Biotechnology Institute, CIBM, Granada, Spain; Biohealth Research Institute (ibs.GRANADA), University Hospitals of Granada, University of Granada, Granada, Spain; Computational and Molecular Evolutionary Biology Research Group, School of life sciences, Faculty of Medicine and Health Sciences, University of Nottingham, Nottingham, UK; Department of Research, Cancer Registry of Norway, Oslo, Norway; Department of Biological Sciences, Dartmouth College, Hanover, USA; Science for Life Laboratory, Department of Molecular Biosciences, The Wenner-Gren Institute, Stockholm University, Stockholm, Sweden; Science for Life Laboratory, Department of Medical Biochemistry and Biophysics, Karolinska Institute, Solna, Sweden; Science for Life Laboratory, Department of Molecular Biosciences, The Wenner-Gren Institute, Stockholm University, Stockholm, Sweden; Science for Life Laboratory, Department of Molecular Biosciences, The Wenner-Gren Institute, Stockholm University, Stockholm, Sweden; Science for Life Laboratory, Department of Microbiology, Tumor and Cell Biology, Karolinska Institute, Solna, Sweden; Science for Life Laboratory, Department of Molecular Biosciences, The Wenner-Gren Institute, Stockholm University, Stockholm, Sweden; Centre for Palaeogenetics, Stockholm, Sweden; Politecnico di Torino, Torino, Italy; Center for Bioinformatics, Department of Informatics, University of Oslo, Oslo, Norway; Department of Tumor Biology, Institute for Cancer Research, The Norwegian Radium Hospital, Oslo University Hospital, Oslo, Norway; Center for Bioinformatics, Department of Informatics, University of Oslo, Oslo, Norway; Department of Genetics, Faculty of Sciences, MNAT Excellence Unit, University of Granada, Granada, Spain; Biotechnology Institute, CIBM, Granada, Spain; Biohealth Research Institute (ibs.GRANADA), University Hospitals of Granada, University of Granada, Granada, Spain; Science for Life Laboratory, Department of Molecular Biosciences, The Wenner-Gren Institute, Stockholm University, Stockholm, Sweden; Department of Biological Sciences, Dartmouth College, Hanover, USA

## Abstract

We describe an update of MirGeneDB, the manually curated microRNA gene database. Adhering to uniform and consistent criteria for microRNA annotation and nomenclature, we substantially expanded MirGeneDB with 30 additional species representing previously missing metazoan phyla such as sponges, jellyfish, rotifers and flatworms. MirGeneDB 2.1 now consists of 75 species spanning over ∼800 million years of animal evolution, and contains a total number of 16 670 microRNAs from 1549 families. Over 6000 microRNAs were added in this update using ∼550 datasets with ∼7.5 billion sequencing reads. By adding new phylogenetically important species, especially those relevant for the study of whole genome duplication events, and through updating evolutionary nodes of origin for many families and genes, we were able to substantially refine our nomenclature system. All changes are traceable in the specifically developed MirGeneDB version tracker. The performance of read-pages is improved and microRNA expression matrices for all tissues and species are now also downloadable. Altogether, this update represents a significant step toward a complete sampling of all major metazoan phyla, and a widely needed foundation for comparative microRNA genomics and transcriptomics studies. MirGeneDB 2.1 is part of RNAcentral and Elixir Norway, publicly and freely available at http://www.mirgenedb.org/.

## INTRODUCTION

With >14 000 publications in 2020 alone, microRNAs, an important group of post-transcriptional gene regulators, continue to dominate the expanding non-coding RNA field. Despite this uninterrupted and increasing interest in microRNAs, a number of serious issues on the quality of microRNA annotations in publicly available repositories (1–14) and the reproducibility of microRNA studies, pervade the field (15). Up to two-thirds of entries for plants (8,16) and animals (10,17) were identified as false-positives, and many animal microRNA complements were found to be incompletely annotated (17). This lack of consistent annotation among microRNA complements across the animal tree of life often resulted in missing data incorrectly interpreted as secondary losses by non-experts (18), raising then questions about the validity of prior studies (19–29) that used microRNAs as phylogenetic markers to explore recalcitrant areas of the metazoan tree (30–33).

To address this and enable comparative microRNA complement analyses across organisms, we previously developed already existing annotation criteria (34) into a next generation sequencing (NGS) strategy to annotate the near-complete microRNA repertoire for any metazoan species (13). Further, we employed a uniform and consistent annotation system for microRNA nomenclature designed to reflect the evolutionary relationships between microRNA genes and family members (10). Using both these annotation and nomenclature systems, we established the microRNA gene database MirGeneDB (https://mirgenedb.org). Initially, this database contained the *bona fide* microRNA complements of only four species, human, mouse, chicken and zebrafish. Forty one additional species were added with the release of MirGeneDB 2.0, including numerous protostome model systems such as *Drosophila* and *Caenorhabditis* (17), as well as several new features including NGS read data representation, isoMir annotations, and known instances of 3′-monouridylation. Nearly 11 000 *bona fide* and consistently named microRNAs constituting 1275 microRNA families were now included into the database, allowing us to confirm the unique phylogenetic utility of microRNAs in animals given their rare losses during evolution (18). Nonetheless, several animal phyla were missing from this version including basal metazoans such as sponge and cnidarian representatives. In addition, several major clades only contained a single representative species including actinopterygian fish, amphibians, lepidosaurs, and chelicerates, greatly limiting the robustness and applicability of the database.

In order to have a truly metazoan-wide microRNA complement, and a more detailed picture of animal microRNA evolution, we present a substantial update of MirGeneDB, MirGeneDB 2.1. This release includes several new animal phyla and 30 new species, totaling now 75 metazoan representatives, and spanning more than 850 million years of animal evolution (35). This addition of phylogenetically interesting and important species allowed us to substantially improve the resolution of phylogenetic node annotation of microRNA genes and families. With >6000 new microRNA genes, for a total of 16 670 entries grouped in 1549 families, ∼150 new sequencing datasets (∼550 in total), and comprising >7.5 billion small RNA sequencing reads, this update of MirGeneDB further strengthens our database for researchers looking for high quality annotations of model and non-model animal species for developmental, homeostatic, disease, and evolutionary analyses.

## EXPANSION OF MirGeneDB

Following our previously described procedure of adding new taxa to MirGeneDB (17), we analyzed more than 150 new datasets that were automatically downloaded and processed using sRNAbench (36) and miRTrace (37), respectively (Supplementary Table S1 for all ∼550 datasets used in MirGeneDB 2.1). These data, along with publicly available genome references, were then used in MirMiner (22) for the annotation of *bona fide* microRNA genes. In a few special cases, including coelacanth, tuatara and the nautilus, although genomic references exist (38–40), no small RNA read data are currently available, and hence the conserved microRNA repertoires of these species were determined using a classical blast approach of closely related species with default settings.

**Figure 1.**
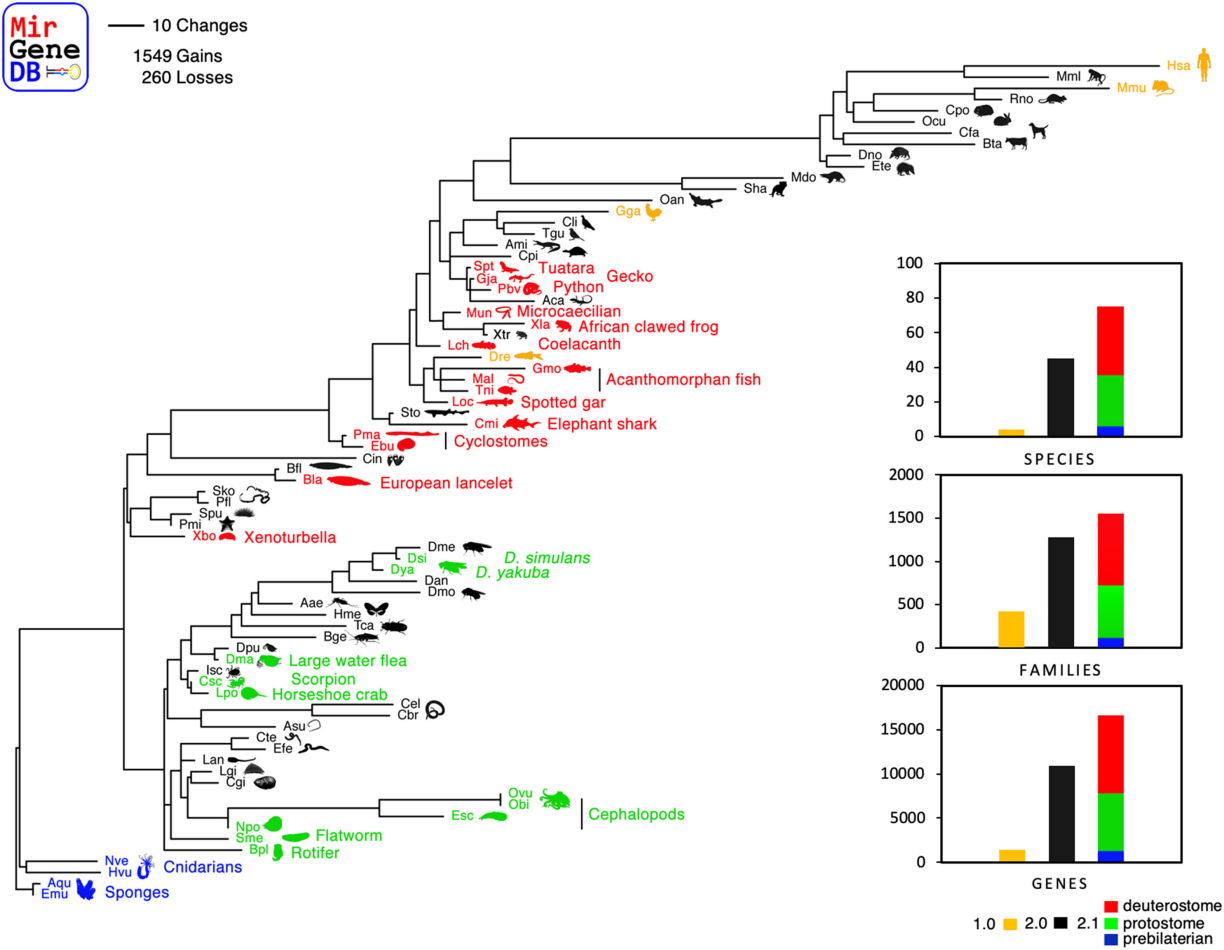
The evolution of the 1549 microRNA families across the 75 metazoan species as annotated in MirGeneDB 2.1 with branch lengths corresponding to the total number of microRNA family-level gains plus family-level losses. Yellow species depict MirGeneDB 1.0 species, black species MirGeneDB 2.0, while blue (pre-bilaterian), green (protostome) and red (deuterostome) species indicate the newly included species in the current update. Inset: the ‘evolution of MirGeneDB’ in terms of number of species, families, and genes, respectively.

To have a truly metazoan-wide microRNA complement in MirGeneDB, we included four non-bilaterian species, the two sponges *Amphimedon queenslandica* and the freshwater sponge *Ephydatia muelleri*, as well as two cnidarians, the freshwater polyp *Hydra vulgaris* and the starlet sea anemone *Nematostella vectensis* (Figure 1, blue). With this inclusion, MirGeneDB now contains the oldest known conserved microRNAs, *Nve-Mir-10*, a member of the eumetazoan MIR-10 family (22,41), with an estimated age of origin likely older than ∼650 million years, and Mir-2019, a sponge-specific microRNA that is likely at least 750 million years old, only around 30 million years younger than the last common ancestor of all living animals and the oldest known eukaryotic microRNA (42).

We further included 11 additional protostome species (6 spiralians, 5 ecdysozoans) to cover more of this incredibly diverse group (Figure 1, green). For spiralian representatives, we added two new metazoan phyla with one representative each: the rotifer *Brachionus plicatilis* and the flatworm *Schmidtea mediterranea*. Further, we substantially expanded the molluscan clade by adding the four cephalopod species *Nautilus pompilius*, the Hawaiian bobtail squid *Euprymna scolopes*, the California two-spot octopus *Octopus bimaculoides* and the common octopus *Octopus vulgaris* (Zolotarov et al., in prep.). For the ecdysozoan node, we added five new arthropod species. We included the Atlantic horseshoe crab *Limulus polyphemus* as well as the Arizona bark scorpion *Centruroides sculpturatus* as two new chelicerate representatives to better characterize the whole genome duplications events found in these lineages (43). We also included the crustacean model system *Daphnia magna* along with two additional *Drosophila* species, *D. simulans* and *D. yakuba*.

Finally, we added 15 new deuterostome species (Figure 1, red) including a new metazoan clade, the Xenoturbellida *Xenoturbella bocki* (Schiffer et al., in prep), a second species of the cephalochordates, the European lancelet *Branchiostoma lanceolatum*, and representatives of the cyclostomes (i.e. jawless vertebrates), the hagfish *Eptatretus burgeri* and the lamprey *Petromyzon marinus* (Pascual-Anaya et al., in prep). We further added a second shark species, the Australian ghostshark *Callorhinchus milii*, and the first representative of the Holostei, the gar *Lepisosteus oculatus*. Furthermore, we added three additional teleost fish, the pufferfish *Tetraodon nigroviridis*, the Atlantic cod *Gadus morhua*, and the Asian swamp eel *Monopterus albus*. We also added the coelacanth *Latimeria chalumnae*, an important species to understand tetrapod evolution. For tetrapods, we added altogether five new species, two amphibians, including the frog model system *Xenopus laevis* and the caecilian *Microcaecilia unicolor*, and three diapsid representatives, including the Burmese python *Python bivittatus*, Schlegel's Japanese gecko *Gekko japonicus* and the tuatara *Sphenodon punctatus*.

The newly included microRNAs are primarily canonical microRNAs, some with 3′-monouridylation including LET-7-P2 members (‘Group 1’ and ‘Group 2’ of Kim et al. (44) for overview). Nonetheless, we added phylogenetically conserved mirtrons, including the placental-specific *Mir-877* and the *Caenorhabditis*-specific *Mir-62*, as well as the Drosha-independent 5' capped microRNAs (‘Group 3’ of Kim *et al.* 2016) of the MIR-320 family, and the DGCR8 exon-encoded MIR-1306. These non-canonical microRNA genes join the only non-canonical microRNA family MIR-451 (‘Group 4’, DICER-independent) that was already included in MirGeneDB 2.0. All non-canonical microRNAs are indicated as such in the ‘Comments’ row.

Despite the additions of these new non-canonical microRNAs, and a complete re-curation of the previously existing microRNA repertoire, the microRNA complements have hardly changed in terms of gene-number (310 previously false negatives were added to and 169 (1.55%) false-positives were removed from the altogether nearly 11 000 genes of MirGeneDB 2.0, see Supplementary Table S2). Therefore, following Bartel (45), most microRNA complements, especially in the case of vertebrates and human in particular, are essentially complete.

## UPDATED NOMENCLATURE IN GNATHOSTOMES AND MirGeneDB-Tracker

**Figure 2.**
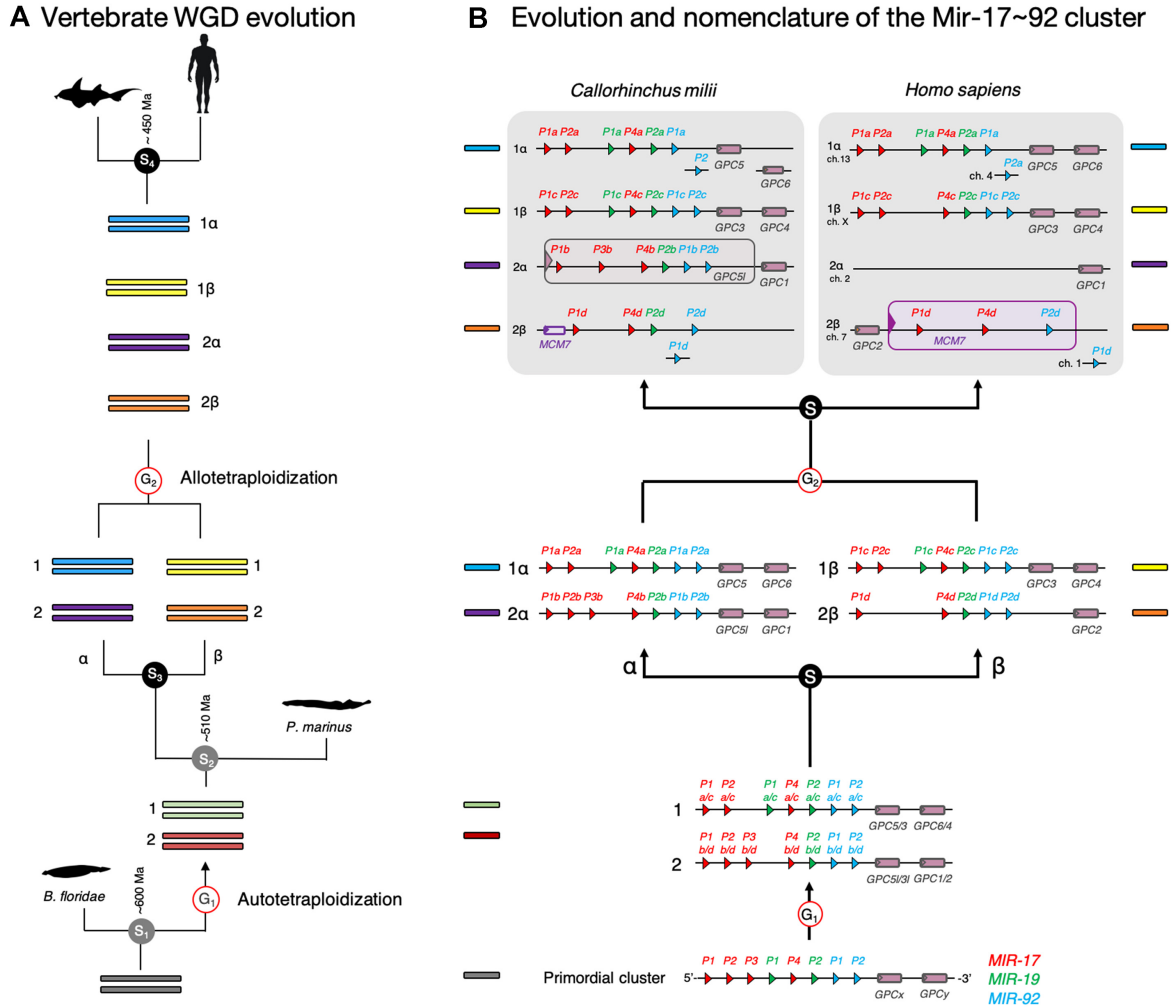
MirGeneDB nomenclature system of gnathostome microRNA genes. (**A**) Model of vertebrate genome evolution adapted from (43). The diploid state of an early chordate ancestor doubled in content (G1) through an autotetraploidy event (38) generating a tetraploid genome. Then, two lineages were generated from a speciation event (S3), α and β. Two species of these lineages then hybridized in an allotetraploidy event (G2), resulting in a single species with an octoploid genome. Sometime soon after this event, around 450 million years ago, the gnathostome LCA evolved and gave rise to the two major extant gnathostome lineages, the Chondrichthyes (the cartilaginous fish) and the Osteichthyes (the bony fish) (S4). (**B**) microRNA gene nomenclature of paralogues, orthologues, ohnologues (genes generated by autotetraploidy events, in this case sub-genomes 1 and 2) and homeologues (gene generated by allotetraploidy events, in this case paralogons α and β) as exemplified by the Mir-17∼92 cluster. See text for details.

Given the inclusion of so many new taxa in MirGeneDB 2.1, we were able to more precisely identify the nodes of origin for numerous microRNA families and genes. This did not affect their nomenclature, only the assigned phylogenetic origin. However, taking advantage of recent insights into the whole genome duplication (WGD) events early in gnathostome history, numerous name changes were also made to reflect the origin of suites of microRNA genes (43). As detailed by Simakov *et al.* (46), vertebrates underwent a single WGD sometime after the split from urochordates, but before the vertebrate last common ancestor (LCA). Then, sometime after this LCA, but before the gnathostome LCA, this genome duplicated again through the hybridization of two species’ genomes. Thus, the early gnathostome genome consisted of four separate paralogous regions or paralogons, each housing a portion of the early gnathostome microRNA repertoire (Figure 2A).

Taking advantage of this historical insight, gnathostome microRNA gene names now reflect the chromosomal history of that particular syntenic region, and thus all genes are consistently named. This nomenclature system is shown in detail for the Mir-17∼92 cluster (Figure 2B). This cluster originally contained 8 genes generated through tandem gene duplication of three distinct families, MIR-17, MIR-19 and MIR-92. The first WGD event resulted in two copies of this cluster followed by the loss of Mir-17-P3a/c on cluster 1, and Mir-19-P1b/d on cluster 2. Then, there was a speciation event resulting in two lineages, what Simakov named α and β. Although there were no losses of any microRNAs in either cluster in the α lineage, the β lineage lost both Mir-17-P2d and Mir-17-P3d. Then, representatives of these two lineages hybridized, bringing together these distinct lineages into a new, now octaploid genome with four separate paralogous regions. After the gnathostome LCA, both the chondrichthyan lineage, as well as the osteichthyan lineage, experienced independent losses, including the entire loss of the 2α cluster in bony fishes. Notice how the new nomenclature system helps not only identity homologous genes with homologous duplication histories in these two taxa (e.g. Mir-17-P1a), but also allows the user to easily identify missing genes like the 2α cluster in bony fishes.

Because originally MirGeneDB identifiers largely just followed miRBase gene names, numerous changes were made in order to go from an effectively random nomenclature system to one that actually tracks the evolutionary history of each microRNA gene generated by these two WGD events. In order to be able to track those name-changes, we have developed a downloadable MirGeneDB version tracker (‘MirGeneDB-tracker’) that will help the user to see the difference between MirGeneDB releases such as name changes, new species, new genes, as well as gene deletions (see Supplementary Table S2).

## IMPROVED WEB-INTERFACE

### Gene-pages

To fit new species, families, genes and read data, while at the same time reducing the computational footprint of our webpage, we included a read page overview page where all data is shown in a summary representation, without resolving individual samples. In a newly developed drop-down menu, these files can be selected independently, but also, if the user prefers, in the classical and computationally more intensive representation.

### Browse and download pages

Navigating through sites with many genes or species, respectively, is now simpler as headers are now frozen when scrolling down through the use of style sheets. Similar to the browse section, rows are now also highlighted in the download-section of the database.

### Count matrix-download

Previously in MirGeneDB 2.0, we had introduced a visual representation of a normalized expression heatmap (RPM) of all samples on our browse-section for each species. This was a popular feature, but we did not provide a downloadable version that some users requested. Therefore, for this release, we provide automatically generated csv-formatted versions of these matrices that are downloadable in the browse & download sections, respectively.

### Information

We have substantially update the information on “Unique structural features of microRNAs” that are the foundation of our annotation system and the corresponding references. MirGeneDB-tracker file can be downloaded on this page. As before, we provide list of false negative accessions, i.e. microRNAs that we detected in read data, but could not locate in either genome-assembly or genomic traces.

## FUTURE DEVELOPMENTS

The establishment of MirGeneDB represents a stable and robust foundation for reproducible microRNA research that overcame a range of curation problems in the microRNA field (10,14,17). With the current update, studies wishing to employ metazoan wide comparative analyses to explore the roles of microRNAs in development and disease (47–49), as well as the evolution of microRNAs and animals themselves (30,50–53), have a much larger range of species available, more easily accessible and with more comprehensive datasets for each species at hand. Our short-term goal will be to focus on specific clades that are either currently not annotated at all, clades that are still poorly represented, or clades for which microRNAs might contribute relevant data for outstanding biological questions. For such groups, we will continue to curate all publicly available data, but we will also generate substantial data ourselves within the MIRevolution framework. We are also looking into the possibilities to incorporate large scale comparative efforts, such as the recent work on all hexapod clades by Ma *et al.* (54) and large genome sequencing initiatives, into our database. The naming of novel microRNAs might become a priority for the next major release depending on the input by the community. Eventually we hope to have curated representatives of all major metazoan clades including multiple species, along with a large number of high quality and low bias datasets from a comprehensive set of organs, tissues, cell types and developmental time points.

## DATA AVAILABILITY

All MirGeneDB data are publicly and freely available under the Creative Commons Zero license. MirGeneDB is part of FAIRsharing.org (55,56). Data is available for bulk download from http://mirgenedb.org/download. Feedback on any aspect of the MirGeneDB database is welcome by email to Bastian.Fromm@uit.no or Kevin.J.Peterson@dartmouth.edu, or via Twitter (@MirGeneDB).

## Supplementary Material

gkab1101_Supplemental_Files
