# Correction to ‘MirGeneDB 2.1: toward a complete sampling of all major animal phyla’

**DOI:** 10.1093/nar/gkac938

**Published:** 2022-10-16

*Nucleic Acids Research*, Volume 50, Issue D1, 7 January 2022, Pages D204–D210, https://doi.org/10.1093/nar/gkab1101

In the originally published version of this manuscript, the following acknowledgement was omitted from the Funding statement:

Funding for open access charge: Stockholm University

This error has been corrected.

